# An Introduction to the Artificial Intelligence-Driven Technology Adoption in Nursing Education Conceptual Framework: A Mixed-Methods Study

**DOI:** 10.3390/nursrep15060184

**Published:** 2025-05-23

**Authors:** Mary Beth Maguire, Anne White

**Affiliations:** 1 Laerdal Medical, Wappingers Falls, NY 12590, USA; 2 WellStar School of Nursing, Kennesaw State University, Kennesaw, GA 30144, USA; awhite@kennesaw.edu

**Keywords:** artificial intelligence, technology adoption, competency-based education, nursing education

## Abstract

**Background/Objectives:** Technological advancements are revolutionizing nursing education by improving precision, patient outcomes, and learning experiences. There is an urgent need for systematic frameworks to help nurse educators effectively integrate advanced technologies into their teaching methods. This manuscript introduces the Artificial Intelligence-Driven Technology Adoption in Nursing Education (AID-TANE) framework and operationalizes its use through a pilot study with undergraduate nursing students. **Methods:** The framework was tested through a convergent mixed-methods pre/post-test study design involving 160 senior-level community health nursing students who participated in an AI-driven educational intervention. Quantitative data were collected using the Facts on Aging quiz, while qualitative data were gathered from a reflective survey. Statistical analyses included paired-sample *t*-tests and a qualitative content analysis. **Results:** The study revealed a statistically significant increase in learners’ knowledge about older adults, with mean scores improving from 33.29 (SD = 5.33) to 36.04 (SD = 6.76) post-intervention (t = 5.05, *p* < 0.001). The qualitative analysis identified four key themes: communication and understanding, patience and empathy, respect for independence, and challenging stereotypes. **Conclusions:** This study found that AI-driven educational tools significantly improved nursing students’ knowledge about older adults and positively influenced their learning experiences. The findings highlight the need for targeted frameworks like AID-TANE to effectively integrate AI into nursing education, ensuring that students are ready for a technologically advanced practice setting.

## 1. Introduction

The healthcare and nursing education landscapes are dramatically transforming, propelled by unprecedented technological advancements. From AI-driven diagnostics to interactive simulations that refine clinical training, innovation redefines how future nursing professionals learn and practice. This era of rapid change presents extraordinary opportunities—enhancing precision, improving patient outcomes, and transforming how learners gain knowledge. As cutting-edge tools merge with human expertise, the future of nursing education is poised to become more dynamic, efficient, and impactful than ever. Now is the moment to embrace progress; however, conceptual frameworks that guide nurse educators on integrating advanced technologies have not kept up with this rapid pace of innovation. This has resulted in an urgent need for a systematic approach to assist nurse educators with the intentional integration of advanced technologies.

Advanced technologies are transforming healthcare by improving precision and patient outcomes. These advanced technologies include the Internet of Things (IoT), artificial intelligence (AI), machine learning (ML), and deep learning (DL). IoT is a connected network of physical devices that collect, exchange, and respond to data over the internet [1]. These technologies have proven effective in assisting patients with Parkinson’s disease to more actively manage their health by providing real-time feedback related to their diet and physical activity, triggering alerts for care teams when malnutrition risk thresholds are crossed [1]. These alerts can improve outcomes through early detection and intervention. Similarly, AI algorithms applied to continuous blood pressure monitoring data are improving the management of hypertension [2]. AI refers to the simulation of human intelligence processes by machines, especially computer systems, to perform tasks such as learning, reasoning, problem-solving, and understanding natural language [3]. The integration of AI models when managing individuals with hypertension can result in more precise management of the disease and a reduction in associated sequelae. Machine learning is a subset of AI that enables systems to learn from data, identify patterns, and make decisions with minimal human intervention [4]. The clinical application of ML includes the computation of large electronic healthcare databases to predict various clinical outcomes, such as pain perception in patients with cognitive impairment, cancer risk, and mortality prediction [5]. Furthermore, DL is inspired by the neural networks of the brain, whereby digital signals (neurons) are connected to create layers, and each layer processes information to find patterns or features [6]. The use of DL is positively influencing the emerging field of Computational Medicine. Computational Medicine is used in medical imaging, electronic health record data mining, and drug development to create more efficient, precise, and accessible care [6]. Advanced technologies have proven transformative in informing and improving healthcare.

The adoption of advanced technologies in healthcare requires nursing education to evolve similarly. However, because there is no framework for integrating advanced technologies into nursing education, the opportunity exists to explore solutions specific to AI. Several AI-driven solutions are available to support nursing education. AI-driven computer-based simulators, AI-driven adaptive learning platforms, and AI-enhanced clinical decision support tools can transform nursing education by creating immersive, adaptive, and personalized learning experiences. AI-driven computer-based simulators provide a lifelike experience to practice clinical communication skills, offering real-time feedback and complex scenarios to enhance decision-making [3]. AI-driven adaptive learning platforms analyze individual progress and tailor educational content to meet students’ specific needs. These platforms adjust the pace, complexity, and focus areas based on learners’ strengths and weaknesses, offering targeted resources and exercises to address gaps in knowledge [7]. AI-enhanced clinical decision support tools integrate real-world applications into the curriculum, teaching learners to interpret patient data and recommend treatments, preparing them for evidence-based care [8]. Together, these technologies foster critical thinking, guided practice, and readiness for tech-driven healthcare environments. Like the impact on healthcare, integrating AI-driven technologies can provide more precise and dynamic instruction to enhance the learning experience for nursing students [9].

Concurrent with the rapid deployment and adoption of AI-driven technologies, nursing is radically shifting to a competency-based educational approach. The move to competency-based education (CBE) in nursing emphasizes measurable outcomes to ensure that learners master essential skills for real-world practice [10]. While conceptual frameworks do not exist to guide the integration of AI-driven technologies into nursing education, frameworks do exist to support CBE. The 5E Instructional Model—Engage, Explore, Explain, Elaborate, and Evaluate—fosters critical thinking and active participation. At the same time, the National College of State Boards of Nursing Clinical Judgment Model (CJM) provides a structured framework to develop and assess critical thinking and decision-making skills [11,12]. These models align seamlessly with AI tools, such as AI-driven simulators and clinical decision support systems, to create personalized, immersive, and feedback-driven learning experiences. Integrating these educational frameworks with AI-driven technologies holds promise for preparing nurses for the complexities of modern healthcare. However, there remains a gap in student-focused conceptual frameworks that help nursing educators effectively integrate AI-driven solutions into their competency-based teaching.

Frameworks do exist to guide the adoption of AI-driven technologies. The United Nations Educational Scientific and Cultural Organization (UNESCO) AI Competency Framework is designed for teachers and is intended to support the integration of AI into educational curricula, ensuring that faculty understand AI technology, its ethical implications, and its societal impact [13]. This model, however, is teacher-focused rather than student-focused. Recently, the Artificial Intelligence in Nursing: Education, Innovation, Collaboration, and Implementation (ALIGN) model has been described [14]. This framework serves to guide the integration of AI technologies into the discipline of nursing; it is not specific to nursing education and does not integrate concepts to support CBE directly [14]. Existing frameworks like UNESCO’s AI Competency Framework and the ALIGN model offer guidance on integrating AI technologies, focusing on ethical understanding and broad applications. Still, they lack student-centered approaches and direct support for CBE in nursing.

**Figure 1:**
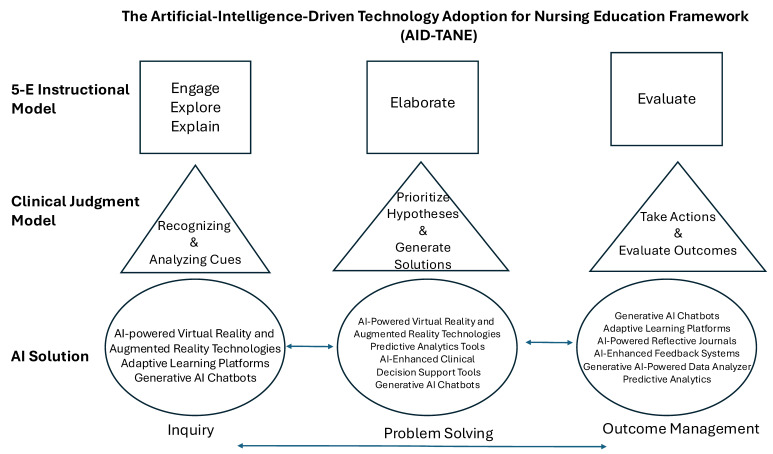
The AID-TANE conceptual framework.

The primary objective of this manuscript was to introduce the AID-TANE conceptual framework, which stands for AI-Driven Technology Adoption for Nursing Education (Figure 1). The secondary objective was to gather evidence to support the framework’s use through a pilot study of undergraduate nursing students using quantitative and qualitative methods. By creating and employing the new framework, nursing education can leverage AI to enhance learning outcomes and prepare learners for future roles.

## 2. Materials and Methods

### 2.1. Aim and Research Question

The research aim was to translate the components of the AID-TANE framework into measurable, actionable elements using a three-phased approach. The research question was as follows: what is the change in undergraduate nursing students’ knowledge of older adults when the AID-TANE framework is used to support a learning event?

### 2.2. Theoretical Framework

The AID-TANE framework combines the 5E educational model [11] and the CJM [12] to guide technology-enhanced instructional design. These models were chosen for their complementary strengths: the 5E Instructional Model promotes active and constructivist learning, while the CJM aligns with professional clinical reasoning and decision-making competencies. Their combination is pedagogically robust, aligning cognitive, metacognitive, and psychomotor domains, thus supporting both skill acquisition and clinical judgment. The AID-TANE conceptual framework creates a comprehensive and dynamic approach to nursing education (Figure 1).

#### 2.2.1. Core Constructs

The first set of core constructs of the AID-TANE is the 5E Instructional Model [11]. This instructional framework promotes active learning and critical thinking through a structured sequence of five phases: Engage, Explore, Explain, Elaborate, and Evaluate (Figure 1). Learners’ interest is piqued in the Engage phase, and their prior knowledge is assessed. The Explore phase involves hands-on activities that allow learners to investigate concepts and develop their understanding. During the Explain phase, learners articulate their understanding, and educators provide further explanations and clarifications. The Elaborate phase encourages learners to apply their knowledge to new situations and extend their learning. Finally, the Evaluate phase involves assessing learners’ understanding and providing feedback to guide future learning. Faculty become guides on the side to coach and support student learning. This framework fosters a more profound comprehension of concepts and helps learners develop essential problem-solving and critical thinking skills.

The AID-TANE framework’s second set of core constructs is inspired by the CJM [12]. The CJM enhances nursing education and practice by outlining nurses’ key steps when making clinical decisions (Figure 1). The model consists of several layers. Layer 1 of the CJM is foundational and focuses on recognizing and analyzing cues. Layer 2 of the CJM concentrates on the clinical reasoning process, which involves identifying and analyzing cues, prioritizing hypotheses, generating solutions, taking action, and evaluating outcomes. This level is essential for understanding how nurses make decisions in real-time clinical situations. Layer 3, however, addresses the cognitive aspects of clinical decision-making, breaking down the process into measurable steps. The connection between Layer 2 and Layer 3 lies in how Layer 2’s clinical reasoning steps are operationalized and measured in Layer 3. Essentially, Layer 3 provides a structured framework to assess and quantify the reasoning processes outlined in Layer 2. The AID-TANE framework integrates Layer 3 of the CJM. The CJM also supports the faculty role of a consultative guide by the side to support CBE.

The AID-TANE framework’s final set of core constructs relates to the types of AI solutions that could assist the educator with developing meaningful learning relative to contemporary nursing practice. The AID-TANE framework synthesizes a learning event into three phases. Phase One is termed the Inquiry phase. The Inquiry phase can be augmented through AI-driven technologies such as immersive VR and AR simulations, adaptive learning platforms, and generative AI chatbots to foster investigation in nursing students by creating personalized, interactive, and engaging learning experiences that encourage exploration and critical thinking. Phase Two is the Problem-Solving phase. Similarly, AI-powered solutions promote nursing students’ problem-solving skills by simulating realistic scenarios, analyzing data, guiding decisions, and fostering inquiry in dynamic learning environments. Phase Three is the Outcome Management phase. AI-powered solutions can promote nursing students’ problem-solving skills in this phase by simulating realistic scenarios, analyzing data, guiding decisions, and fostering inquiry in dynamic learning environments.

The movement between the phases acknowledges that learners may progress and regress as they navigate from Inquiry to Outcome Management. The Inquiry phase incorporates the Engage, Explore, and Explain activities of the 5E framework and the Form Hypothesis phase of the CJM. The Problem-Solving phase incorporates the Elaborate phase of the 5E framework and the Refine Hypothesis of the CJM. The Outcome Management phase incorporates the Evaluate phase of the 5E framework and the Evaluation phase of the CJM. Together, these evaluation processes promote the mastery of skills and critical thinking essential for nursing practice, reinforcing the competency-based approach’s focus on real-world readiness and measurable achievement. In an era of the rapid development and deployment of AI solutions, the AID-TANE framework provides a structure for integrating the technologies within the educational continuum.

The first two layers of the AID-TANE framework are student-focused and encourage active learner engagement to promote clearly defined, measurable skills and knowledge essential for safe, effective clinical practice. The last layer provides guidance to nurse educators for potential AI solution integration. The framework helps nurse educators integrate AI-driven technologies into teaching activities at any phase of the educational continuum in a move toward teaching to competence. By breaking down the decision-making process into measurable steps, the AID-TANE framework can practically assist educators with the intentional integration of AI solutions to systematically guide learners by connecting theoretical knowledge to practical clinical care.

#### 2.2.2. Application to Nursing Education

The AID-TANE conceptual framework supports nursing education by incorporating the 5E educational framework [11] with the CJM [12] and integrating advanced AI technologies to support CBE. The 5E model fosters active learning, critical thinking, and knowledge application through its structured phases—Engage, Explore, Explain, Elaborate, and Evaluate. The CJM enhances clinical decision-making by guiding learners through recognizing patient cues, prioritizing diagnoses, implementing care plans, and evaluating outcomes. AI technologies offer the opportunity to personalize learning, simulate clinical scenarios, and equip learners for a technology-driven healthcare environment. Adopting AI technologies in teaching creates a dynamic, future-ready educational approach that builds aspiring nurses’ problem-solving, thinking, and adaptability skills.

#### 2.2.3. Ethical Alignment

Ethical alignment must be considered when utilizing AI technologies in nursing education. On the positive side, AI can enhance personalized learning, support equitable access to educational resources, and improve the quality of nursing training through realistic simulations and data-driven feedback [15]. However, ethical concerns arise regarding data privacy, the risk of over-reliance on AI tools, and the potential for bias in AI algorithms [15]. Utilization of the framework must prioritize safeguarding student and patient data, ensuring transparency in AI processes, and teaching students critical thinking to independently validate AI-generated insights. Additionally, ethical training should be integrated into the curricula to prepare future nurses to use AI responsibly and maintain patient-centered care. By acknowledging these issues when using the AID-TANE framework, faculty can promote the ethical adoption of AI while maximizing its benefits in nursing education.

#### 2.2.4. Organizational Readiness

Organizational readiness is critical in successfully implementing the AID-TANE framework in nursing education. Adopting AI technologies and associated frameworks involves assessing the institution’s capacity to adopt new technologies, integrating AI into existing educational models, and addressing ethical concerns [15]. Key considerations include ensuring that faculty and staff have the necessary training and resources to effectively use AI tools and frameworks like the 5E model and the CJM. Institutions must also invest in robust infrastructure to support AI technologies while maintaining data privacy and security measures. Additionally, fostering a culture that values innovation, ethical practice, and adaptability is essential. By proactively addressing these factors, organizations can create an environment that supports meaningful and sustainable nursing education advancements.

### 2.3. Design

To operationalize the AID-TANE framework, the researchers utilized a convergent mixed-methods pre/post-test study design to assess learners’ knowledge of older adults. Integrating quantitative and qualitative data adhered to a convergent mixed-methods design, where both data types were collected and analyzed separately yet concurrently. This approach aimed to provide a comprehensive understanding of the impact of an AI-driven educational (AIED) intervention on nursing students’ knowledge of older adults. The study focused on senior-level community health nursing students in a southeastern United States baccalaureate program. The program offers traditional and accelerated Bachelor of Science in Nursing programs, with the traditional program spanning five semesters and the accelerated program covering four consecutive semesters for learners with prior degrees. The curriculum integrates humanities, biological and social sciences, and theoretical and clinical practice to prepare learners for professional nursing. The instructional content includes over 800 clinical hours, psychomotor skills labs, and simulation experiences, emphasizing evidence-based learning. The delivery modes are primarily on-campus and in-person modes, with no part-time options available.

### 2.4. Sample Demographics

All students enrolled in the senior-level community health nursing course were included and received eight hours of simulation credit for the experience. The cohort included both traditional and accelerated students.

### 2.5. Inclusion/Exclusion Criteria

The single eligibility criterion for this study was enrollment in the community health nursing course as a matriculated baccalaureate nursing student.

### 2.6. Instruments

#### 2.6.1. Facts on Aging

The Facts on Aging quiz [16] was used to measure the knowledge of older adults gained through the learning activities [Supplemental File S1]. The survey was first introduced by Palmore in 1977 and was last revised by Breytspraak and Badura in 2015. The Facts on Aging quiz consists of 50 true/false questions to assess the knowledge of older adults. The questions are scored 1 point for a correct answer and 0 points for an incorrect answer, with a total possible score of 50 points. A higher score indicates higher knowledge. The questions are related to the mental health, physical health, lifestyle factors, and life transitions of older adults. The survey scores provide perspectives regarding aging and clear statistics associated with the realities of aging [17].

#### 2.6.2. Guided Reflection Survey

The authors created a three-question, open-ended guided reflection survey to gain insights into the learners’ impressions of the learning event. The three questions aimed to understand the impact on knowledge, skills, and attitudes after participating in the AIED (Table 1). The questions were as follows: Describe what you learned regarding working with older adults through participation in the simulation activity. Identify three strategies you will use when working with older adults in your future nursing practice. Describe three ways your attitude toward working with older adults has been impacted by participation in the simulation. For this study, only the first item, which focused on knowledge gained, was analyzed. This decision was based on the study’s primary aim of evaluating knowledge acquisition rather than skill-based or affective outcomes. Future studies will explore the remaining questions.

### 2.7. AI-Driven Technology Employed

Two AI-driven solutions were utilized in this study. First, the PCS Spark AI-driven computer-based simulator provided an immersive platform for the students to practice clinical decision-making in a safe, controlled environment [18]. This tool offered dynamic, real-time scenarios programmed by the faculty with assistance from the product specialist. The simulator was trained to respond according to the learning objectives and outcomes of the simulations. The National League for Nursing Advancing Care Excellence for Seniors resources for Millie Larsen [19] were used to design the case, and adherence to healthcare simulation standards of best practice was employed in the scenario design [20]. The learners engaged with the simulator by communicating various questions and answers via a computer microphone; these inputs served as prompts for the simulator to analyze and respond. The simulator adapted to the learners’ prompts, fostering critical thinking and competency development.

Second, a qualitative data analysis of the guided reflection surveys using the ATLAS.ti program enabled a structured examination of the students’ reflective practices [21]. Text responses to a guided reflection survey were uploaded, and the program was prompted to identify themes and highlight text to support the associated themes. By identifying patterns and themes in the survey responses, ATLAS.ti facilitated the evaluation of the depth of student learning and self-assessment, supporting continuous improvement in instructional strategies and learner outcomes. These technologies represented a reasonable sample of AI-driven solutions to operationalize the AID-TANE framework.

### 2.8. Statistical Methods

#### 2.8.1. Quantitative Analysis

Quantitative data were collected using the Facts on Aging quiz [16] administered to the learners before and after the intervention. This survey provided numerical data on the learners’ knowledge, which were analyzed using paired-sample *t*-tests to determine any statistically significant changes in knowledge pre- and post-intervention. Normality was checked using a quantile–quantile plot and the Shapiro–Wilk test. The *p*-value and effect size are reported to provide context for the results. All statistical analyses were performed using R version 4.3.2 [22].

#### 2.8.2. Qualitative Analysis

For the qualitative data analysis, ATLAS.ti [21] software (version 23) organized and analyzed the text responses to question one of the guided reflection surveys. The data were systematically coded using generative AI to identify key themes, and a hierarchical coding system was developed to categorize recurring patterns. Advanced search and query functions were used to explore relationships between codes and uncover co-occurring themes. The researchers conducted coder validation to ensure reliability by cross-verifying interpretations of the data. Throughout the analysis process, memos were written to capture reflections and insights. Finally, reports were generated and exported to present the findings clearly and efficiently.

#### 2.8.3. Triangulation Methods

The triangulation of results was employed to enhance the credibility and depth of the study’s findings. Data from both qualitative and quantitative sources were compared and integrated to identify areas of convergence, complementarity, and divergence. This approach allowed for a more comprehensive understanding of the phenomenon under investigation, ensuring that the interpretations were well supported across multiple forms of evidence.

### 2.9. Intervention

Table 1 identifies the learning activities included in the intervention. Once the learners completed the pre-simulation work, they engaged with the simulator. Due to the virtual nature of this activity, the participants were given one week to finish the assigned tasks. Before starting the encounter, the learners were introduced to the virtual patient simulator [18]. This simulator was an AI-driven, computer-based platform. Each student had an individual account that allowed them to access the simulation at any time and repeat it as often as necessary. By using the computer microphone, the learners engaged in conversation with Millie Larson, who is part of the National League for Nursing’s Advancing Care Excellence for Seniors resources [19], to complete an abbreviated Outcome and Assessment Information Set Start of Care (OASIS SOC) [23]. The OASIS SOC is an assessment tool used to initiate home healthcare for older adults, and it is also utilized for medical insurance reimbursement. A nurse’s OASIS SOC assessment is regularly discussed in interdisciplinary team meetings to establish a comprehensive treatment plan. However, for this learning activity, the learners were asked to compose a draft email to an interdisciplinary team, summarize the patient case, and provide recommendations for future care to simulate an interdisciplinary conference.

### 2.10. Ethical Considerations

The university’s Institutional Review Board (IRB) approved the study. It adhered to ethical principles, including independence, autonomy, confidentiality, and respect for participants. The participants were informed that completing the learning activity was mandatory for the course. However, informed consent was obtained from all participants, who were made aware of their right to withdraw without impacting their course standing. This approach ensured that, while the educational activity was mandatory, the learners’ involvement in the study adhered to ethical standards, as approved by the university’s IRB. The participants were treated respectfully throughout the study and acknowledged for their contributions. All study-related data were de-identified and securely stored on password-protected computers.

Given the integration of AI technologies in instructional design, additional ethical considerations were addressed. The learners were explicitly informed of the use of generative AI (e.g., ATLAS.ti and AI-based simulators) and provided consent for participation in digital learning environments that utilize algorithmic feedback. The instructional team ensured that the learners received foundational instruction in AI literacy, including the limitations and potential biases of large language models. Additionally, all platforms used in the intervention adhered to the Family Educational Rights and Privacy Act and institutional privacy protocols.

Ethical training was embedded in pre-simulation instruction, where the learners discussed algorithmic transparency, data security, digital professionalism, and the nurse’s role in validating AI-derived information with participants. This multifaceted approach aimed to foster an ethical, reflective use of AI in clinical education, supporting student autonomy and technological fluency.

## 3. Results

### 3.1. Sociodemographics of the Sample

One hundred sixty students participated in this study. Of these participants, 144 (90%) identified as female, and 16 (10%) identified as male. Additionally, 79 (49%) were aged 18–24 years, 62 (39%) were aged between 25 and 34 years, 11 (7%) were aged between 35 and 44 years, 7 (4%) were aged between 45 and 54 years, and 1 (<1%) was aged between 55 and 64 years. Demographic data were collected from the course registration list.

### 3.2. Instrument Reliability

The Facts on Aging quiz has been studied, and the current literature supports its validity and reliability. Davis and Zechner [24] used the quiz with nursing students before and after interacting with older adults at a senior center. The researchers reported an increase in mean scores (t = −5.3, *p* = 0.000). Therefore, evidence exists to reinforce the quiz’s utility as an acceptable educational tool for assessing knowledge about aging.

### 3.3. Mean Differences in Scores

This study aimed to determine whether an AIED event influenced community health nursing learners’ knowledge regarding older adults. Knowledge was assessed before and after the event using the Facts on Aging quiz [16], where the total number of correct responses was tabulated at each time point for each student participant. Only learners who completed both the pre-test and the post-test were included in the analysis (*n* = 160). The results of a paired *t*-test indicate that knowledge improved from before the encounter (*M* = 33.29, *SD* = 5.33) to after the encounter (*M* = 36.04, *SD* = 6.76), and this modest improvement was determined to be statistically significant (*t* = 5.05, *p* < 0.001). The effect size, calculated using Cohen’s *d*, was 0.40, indicating a moderate practical significance of the intervention.

### 3.4. Qualitative Results

The data from the reflective journals were analyzed using a deductive content analysis, revealing four key themes: communication and understanding, patience and empathy, respect for independence, and challenging stereotypes (Table 2). Many learners highlighted the importance of effective communication and understanding when interacting with older adults, especially after the simulated encounter with Millie Larsen in her home. They emphasized adapting their communication styles to ensure clarity and comprehension. The learners acknowledged the value of being patient and empathetic during interactions with older adults, recognizing that these individuals might require more time to respond and that demonstrating empathy can significantly enhance the quality of care. Additionally, the learners observed that older adults value their independence and autonomy, learning to respect and support this independence while providing necessary care. Finally, the learners understood that older adults often possess greater capabilities and resilience than initially thought, challenging their preconceived notions.

### 3.5. Triangulation of Quantitative and Qualitative Findings

The quantitative findings demonstrate a statistically significant improvement in the learners’ knowledge about older adults following the AIED event, with a moderate effect size (Cohen’s *d* = 0.40). This suggests that the encounter had a meaningful educational impact. These gains in factual knowledge were further contextualized and deepened by the qualitative analysis of the learners’ reflective journals, which revealed four key themes: communication and understanding, patience and empathy, respect for independence, and challenging stereotypes.

Together, the two strands of data suggest that the AIED experience effectively increased the learners’ cognitive knowledge, as measured by the Facts on Aging quiz, and that it was transformative in shaping their attitudes and clinical reasoning. For example, the improved communication strategies highlighted in the reflections (e.g., avoiding medical jargon and adjusting speech pace) align with a deeper understanding of older adults’ needs, an insight not captured by the quiz alone. Likewise, themes such as patience, empathy, and the value of independence reflect the internalization of learning outcomes that extend beyond factual recall and demonstrate the development of affective and relational competencies.

The qualitative themes also support the quantitative outcome by illustrating how the learners translated their increased knowledge into meaningful attitudes and behaviors during simulated encounters. The convergence of findings across the quantitative and qualitative strands supports the overall effectiveness of the AIED intervention in enhancing both knowledge and the humanistic dimensions of caring for older adults.

## 4. Discussion

This study explored the impact of an AIED on community health nursing students’ knowledge regarding older adults and their perceptions of the learning experience to support the use of the AID-TANE framework. The findings demonstrate that the AIED led to a statistically significant improvement in factual knowledge, with a moderate effect size (Cohen’s *d* = 0.40). In addition, a qualitative analysis of the learners’ reflections provided insights into how advancing technology using an AI-driven tool and competency-based educational strategies influenced their learning. The integration of findings confirms the AIED intervention’s effectiveness in improving knowledge and humanistic care for older adults.

### 4.1. Advancing Technology

The observed improvement in the learners’ knowledge after the AIED intervention reflects the broader trend of technological innovation transforming healthcare and education [1,6]. The learners’ qualitative feedback emphasized how interactive, real-time experiences made learning more engaging and meaningful. Several learners commented that the AIED platform created a “dynamic and responsive learning environment”, echoing the literature that describes how integrating technologies such as IoT and DL enhances feedback loops and early intervention in healthcare [1,6]. This supports the idea that leveraging advanced technologies within nursing curricula can similarly transform educational delivery, making learning more adaptive, precise, and impactful.

### 4.2. Incorporation of AI-Driven Technologies to Prepare Learners for Evidence-Based Care

The findings also support the thesis that AI-driven technologies can prepare nursing students for evidence-based practice, as Guraya [3] and Chew and Ngiam [8] discussed. The learners reflected positively on their ability to apply real-time data interpretation and evidence-based guidelines during the AIED event. Comments such as feeling “better prepared to use patient data to guide clinical decisions” mirror the promise of AI-driven simulators in enhancing student readiness for real-world evidence-based care [3,8]. This alignment between educational intervention and clinical practice skills is crucial as healthcare increasingly relies on AI to drive precision in diagnostics, management, and treatment decisions [2,5].

### 4.3. Advancing Competency-Based Education Through AI-Driven Technologies

The results also connect with the broader shift toward competency-based education (CBE) models in nursing, emphasizing measurable, practice-ready skills [10]. The moderate but significant increase in knowledge after the AIED intervention, combined with the learners’ reflections on how the event helped them recognize personal strengths and areas for growth, suggests that AI-driven technologies can effectively support CBE objectives. Specifically, the structured, feedback-driven approach of the AIED event parallels existing educational frameworks like the 5E model and the CJM [11,12], both of which aim to promote critical thinking and decision-making. Moreover, the learners’ appreciation for targeted, real-time feedback supports the idea that the integration of AI tools can personalize learning pathways, a key advantage identified by Linds et al. [7].

Additionally, the qualitative responses of the senior-level baccalaureate nursing students reflect a basic understanding regarding caring for older adults. These findings confirm evidence from a systematic review and meta-analysis that analyzed nursing students’ willingness to care for older adults [25]. The study revealed that only 49% of nursing students were willing to work with older populations, with prior experience living or caring for older adults being a significant predictor of willingness. The student comments are further supported by research highlighting that working with older adults is often seen as unattractive to healthcare students, including nursing students, which limits their exposure and interest in this population [26]. The findings of this study suggest that AI-driven simulators can increase experience with older adults and positively influence learners’ knowledge of this population’s unique needs and challenges.

### 4.4. The Need for Targeted Frameworks

While frameworks such as UNESCO’s AI Competency Framework and the ALIGN model [14] offer broader guidance for AI adoption, they do not directly address competency-based nursing education or focus on student-centered outcomes. The positive results of this study underscore the need for targeted frameworks like AID-TANE, which align the integration of AI technologies with measurable competencies in nursing education. Targeted frameworks for AI integration positively influence nursing education by aligning advanced technologies with measurable competencies, fostering personalized learning experiences, and enhancing critical thinking and decision-making skills [27]. This study supports using the AID-TANE framework to integrate technology to advance learners’ knowledge of the care of older adults. Appreciation for the student-focused framework was evident in responses that reflected that the learners were able to rehearse their communication with the AI-driven simulator and were given real-time feedback that was immediate and targeted to their individual performance. The convenience of the computer-based simulator allowed the participants to engage when and where it was convenient for them. These frameworks ensure that AI-driven technologies effectively prepare nursing students for real-world challenges. They also promote inclusivity and equity by tailoring education to diverse learner needs, bridging the gap between academic preparation and clinical practice.

### 4.5. Limitations

Several limitations related to this study warrant further exploration and consideration. First, a single-site research design was utilized, which limits the generalizability. Next, the study employed a single AI-driven simulation along with several additional learning activities at one point in the nursing curriculum. This may have influenced the positive study outcomes. Additionally, the novelty of AI adoption in nursing education presents challenges. The lack of established benchmarks or standardized methodologies for evaluating AI’s role introduces potential biases in data interpretation. External variables, such as faculty engagement with the technology or prior exposure to AI concepts, could have influenced the outcomes, further complicating the attribution of the results solely to the AI-driven intervention. Furthermore, the reliance on self-reported data in evaluating the impact of guided reflection surveys and AI-powered tools introduces a potential bias, as responses may reflect perceived rather than actual gains in competency. Finally, while the study underscores the potential of the AID-TANE framework, continued testing with diverse AI-driven technologies is necessary to gather robust evidence that supports its widespread adoption and ensures its adaptability to varied educational settings.

### 4.6. Future Research

Future research should explore the scalability of the AID-TANE framework across diverse educational settings and its adaptability to various nursing curricula. Also, future research should examine avenues for faculty to incorporate AI-driven solutions using the AID-TANE framework at different points throughout the curriculum. This is essential to support using advanced technologies to support learning outcomes. Additionally, future research should incorporate triangulation by using a combination of quantitative measures, objective assessments, and qualitative insights to validate outcomes using the AID-TANE framework. Finally, the stability of the outputs of AI-driven solutions should be continuously monitored to ensure that the algorithms are maturing properly with minimal bias and effect in influencing outcomes beyond those intended.

### 4.7. Practical Implications

The results of this study support the use of the AID-TANE framework in nursing education, highlighting its potential to transform learning environments through the integration of AI technologies. The findings support using AI-driven tools to promote CBE, ensuring that nursing students achieve measurable outcomes in critical areas such as inquiry, problem-solving, and outcome management. A conceptual framework like AID-TANE provides a structured approach to enhance learning outcomes, foster innovation, and bridge the gap between academic preparation and clinical practice.

## 5. Conclusions

During the era of unprecedented technological change in nursing education, extraordinary opportunities are available to enhance precision, improve patient outcomes, and transform how learners gain knowledge using AI-driven technologies. Scholars are called to rigorously explore the AID-TANE framework across diverse disciplines and institutions, refine its methodologies, address existing limitations, and unlock its full potential as a cornerstone of AI integration into competency-based nursing education. This is not merely a recommendation but an urgent invitation to advance the field with evidence-driven insights that will shape the future of AI integration into nursing education and healthcare practices.

## Figures and Tables

**Table 1:** Study summary.

Intervention	AI Technology Integration	Data Collection Method
Pre-intervention		
Assessment: Facts on Aging survey	* AI-Based tools for data analysis	Online survey
Inquiry phase		
1. Pre-class readings	* AI-driven learning platforms	N/A
2. Ninety-minute interactive classroom session	* AI-driven analytic tools	N/A
3. Pre-simulation electronic medical record review	* Generative AI chatbot	N/A
4. Pre-simulation knowledge assessment of simulated patient information	* AI-based tools for data analysis	Online survey
Problem-Solving phase		
1. Virtual simulated patient interview	AI-powered virtual reality simulator	N/A
2. Completion of the OASIS form	N/A	Online survey
3. Create report email to multidisciplinary team	* Generative AI chatbot	Online document
Outcome Management phase		
1. Guided reflection survey/debrief	Generative AI-powered data analysis tool	Online survey
2. NLN simulation design scale completion	* AI-based tools for data analysis	Online survey
Post-intervention assessment:		
Facts on Aging survey	* AI-based tools for data analysis	Online survey

* Indicates AI solution that could be employed.

**Table 2:** Qualitative findings.

Qualitative Theme	Participant Comment
Communication and Understanding	“While speaking to older adults, it is important to speak slowly and enunciate words properly. Be clear and concise and avoid unnecessary medical jargon.” “During the simulation activity I learned to try and avoid medical jargon and speak at the comprehension level of the patient to help minimize confusion.” “I learned that older adults do not like for you to ask them a bunch of questions at one time. It is best to ask simple and direct questions.”
Patience and Empathy	“I learned that when you are working with older adults, you have to take your time in obtaining a detailed and accurate health report.” “It is important to have patience and to actively listen to older patients.” “I learned that you have to be patient and take your time when interviewing older adults. It may take them longer to answer the questions; however, they are still willing to learn and grow like the rest of us.” “Give older adults more time to formulate responses. Sometimes you must clarify what you are asking.”
Respect for Independence	“Older adults still desire independence and want to maintain a certain level of independence for as long as possible.” “Older adults are quite capable of doing things on their own but might require some assistance with different tasks.” “Older adults want to maintain their independence. They do not want to have to rely on others and want to be able to maintain their previous pace, activities, etc.” “Older adults like to maintain their independence and reiterate that they can do things independently.”
Challenging Stereotypes	“Not all elderly people need help, most of them can do a lot for themselves and may only need help with minor things.” “Older adults are still able to do the vast majority of their ADLs and leisure activities on their own.” “Older adults are capable of answering questions and providing additional information just as easily as any other patient.”

## Data Availability

The data presented in this study are available on request from the corresponding author.

## Supplementary Materials

The following supporting information can be downloaded at: https://www.mdpi.com/article/10.3390/nursrep15060184/s1, File S1: Facts on Aging Quiz.

## Public Involvement Statement

There was no public involvement in any aspect of this research.

## Guidelines and Standards Statement

This manuscript was drafted against the SQUIRE-ED (Standards for Quality Improvement Reporting Excellence in Education: Publication Guidelines for Educational Improvement) for qualitative research.
