# Supplementary material for: An Introduction to the Artificial Intelligence-Driven Technology Adoption in Nursing Education Conceptual Framework: A Mixed-Methods Study
Source: Nurs Rep. 2025 May 23;15(6):184. doi: 10.3390/nursrep15060184 (PMC12196197; doi:10.3390/nursrep15060184)
Supplement: Supplementary file 1 [file nursrep-15-00184-s001.zip › nursrep-3590937-supplementary.pdf]

Facts on Aging Quiz

Revised by Linda Breytspraak[1], Ph.D., and Lynn Badura, B.A., Grad. Gerontology Certificate
Gerontology Program
University of Missouri--Kansas City
2015

There have been a number of versions of quizzes on aging, patterned after Erdman Palmore's landmark "Facts on Aging Quiz" that appeared in two issues of *The Gerontologist* (1977; 1981). The initial version developed at UMKC was authored by Linda Breytspraak, Ph.D., Burton Halpert, Ph.D., and Liz Kendall, M.A. The current revision of that initial version was authored by Linday Breytspraak, PhD, and Lynn Badura, B.A., Graduate Certificate in Gerontology.

About half the items in the current quiz are similar or identical to Palmore's. The other half represent issues that have received more attention since his quiz was developed or were judged by the authors to be of significant interest now. This 2015 revision has all the same questions as the original version with a few small wording changes in several items. The authors have drawn on current research and gerontological and geriatric texts to answer the questions. We provide a reference list, divided into (1) general sources and (2) sources used to document data or specific trends discussed in answers to particular questions.

The authors of the 2015 version grant permission for anyone to use the Facts on Aging Quiz for educational purposes as long as credit is given using the following citation:

Breytspraak, L. & Badura, L. (2015). *Facts on Aging Quiz* (revised; based on Palmore (1977; 1981)). Retrieved from http://info.umkc.edu/aging/quiz/.

[1] Contact information for first author: breytspraakl@umkc.edu

Facts on Aging Quiz

T F 1. The majority of old people (past 65 years) have Alzheimer's disease.

T F 2. As people grow older, their intelligence declines significantly.

T F 3. It is very difficult for older adults to learn new things.

T F 4. Personality changes with age.

T F 5. Memory loss is a normal part of aging.

T F 6. As adults grow older, reaction time increases.

T F 7. Clinical depression occurs more frequently in older than younger people.

T F 8. Older adults are at risk for HIV/AIDS.

T F 9. Alcoholism and alcohol abuse are significantly greater problems in the adult population over age 65 than that under age 65.

T F 10. Older adults have more trouble sleeping than younger adults do.

T F 11. Older adults have the highest suicide rate of any age group.

T F 12. High blood pressure increases with age.

T F 13. Older people perspire less, so they are more likely to suffer from hyperthermia.

T F 14. All women develop osteoporosis as they age.

T F 15. A person's height tends to decline in old age.

T F 16. Physical strength declines in old age.

T F 17. Most old people lose interest in and capacity for sexual relations.

T F 18. Bladder capacity decreases with age, which leads to frequent urination.

T F 19. Kidney function is not affected by age.

T F 20. Increased problems with constipation represent a normal change as people get older.

T F 21. All five senses tend to decline with age.

T F 22. As people live longer, they face fewer acute conditions and more chronic health conditions.

T F 23. Retirement is often detrimental to health--i.e., people frequently seem to become ill or die soon after retirement.

T F 24. Older adults are less anxious about death than are younger and middle-aged adults.

T F 25. People 65 years of age and older currently make up about 20% of the U.S. population.

T F 26. Most older people are living in nursing homes.

T F 27. The modern family no longer takes care of its elderly.

T F 28. The life expectancy of men at age 65 is about the same as that of women.

T F 29. Remaining life expectancy of blacks at age 85 is about the same as whites.

T F 30. Social Security benefits automatically increase with inflation.

T F 31. Living below or near the poverty level is no longer a significant problem for most older Americans.

T F 32. Most older drivers are quite capable of safely operating a motor vehicle.

T F 33. Older workers cannot work as effectively as younger workers.

T F 34. Most old people are set in their ways and unable to change.

T F 35. The majority of old people are bored.

T F 36. In general, most old people are pretty much alike.

T F 37. Older adults (65+) have higher rates of criminal victimization than adults under 65 do.

T F 38. Older people tend to become more spiritual as they grow older.

T F 39. Older adults (65+) are more fearful of crime than are persons under 65.

T F 40. Older people do not adapt as well as younger age groups when they relocate to a new environment.

T F 41. Participation in volunteering through organizations (e.g., churches and clubs) tends to decline among older adults.

T F 42. Older people are much happier if they are allowed to disengage from society.

T F 43. Geriatrics is a specialty in American medicine.

T F 44. All medical schools now require students to take courses in geriatrics and gerontology.

T F 45. Abuse of older adults is not a significant problem in the U.S.

T F 46. Grandparents today take less responsibility for rearing grandchildren than ever before.

T F 47. Older persons take longer to recover from physical and psychological stress.

T F 48. Most older adults consider their health to be good or excellent.

T F 49. Older females exhibit better health care practices than older males.

T F 50. Research has shown that old age truly begins at 65.

Answers to
Facts on Aging Quiz

1. **The majority of old people (past 65 years) have Alzheimer's disease.**

   False. According to the 2014 Alzheimer's Disease Facts and Figures Report published by the Alzheimer's Association, one in nine people 65 and older (11%) have Alzheimer's disease. About one‐third of people age 85 and older (32%) have Alzheimer's disease. Of those with Alzheimer's disease, the vast majority (82%) are age 75 or older.

2. **As people grow older, their intelligence declines significantly.**

   False. Although there are some circumstances where the statement may hold true, current research evidence suggests that intellectual performance in healthy individuals holds up well into old age. The average magnitude of intellectual decline is typically small in the 60s and 70s and is probably of little significance for competent behavior. There is more average decline for most abilities observed once the 80s are reached, although even in this age range there are substantial individual differences. Little or no decline appears to be associated with being free of cardiovascular disease, little decline in perceptual speed, at least average socioeconomic status, a stimulating and engaged lifestyle, and having flexible attitudes and behaviors at mid‐life. The good news is that research data now indicate that this is a life stage programmed for plasticity and the development of unique capacities and that intellectual decline can be modified by life‐style interventions, such as physical activity, a healthy diet, mental stimulation, and social interaction.

3. **It is very difficult for older adults to learn new things.**

   False. Although learning performance tends on average to decline with age, all age groups can learn. Research studies have shown that learning performances can be improved with instructions and practice, extra time to learn information or skills, and relevance of the learning task to interests and expertise. It is well established that those who regularly practice their learning skills maintain their learning efficiency over their life span.

4. **Personality changes with age.**

   False. Personality remains consistent in men and women throughout life. Personality impacts roles and life satisfaction. Particular traits in youth and middle age will not only persist but may be more pronounced in later life.

5. **Memory loss is a normal part of aging.**

   True. As one ages there is modest memory loss, primarily short--term memory (recent events). Older adults are more likely to retain past or new information that is based on knowledge acquired or builds upon their life course or events. Retrieval of information may slow with age. The causes of these changes are unknown, but may include stress, loss, physical disease, medication effects, depression, and age--related brain changes. Lack of attention, fatigue, hearing loss, and misunderstanding are among factors impacting memory loss in persons of all ages. Strategies such as activity and exercise, association, visualization, environmental cueing, organization by category and connection to a place may help to prompt memory.

6. **As adults grow older, reaction time increases.**

   True. Reaction time is the interval that elapses between the onset of a stimulus and the completion of a motor response, such as hitting the brake pedal of a car when the traffic light turns yellow or red. When processing ordinary stimuli, adults do show large increases in response time with increasing age.

7. **Clinical depression occurs more frequently in older than younger people.**

   False. There is no evidence that depression occurs more often in older adults than younger groups, and it should not be considered a normal part of aging. However, it is the most common mental health problem of older adults. Depression may vary from feeling "blue" from grief over a loss to a diagnosis of clinical depression by the DSM--5 criteria. Accurate diagnosis and treatment options are often hindered by the resistance to mental health intervention and by situational depression in older adults as they react to isolation, role change, illness, and medication effects.

8. **Older adults are at risk for HIV/AIDS.**

   True. Americans aged 50 and older have many of the same HIV risk factors as younger Americans. According to the Centers for Disease Control and Prevention, persons aged 55 and older accounted for 26% of the estimated 1.2 million people living with HIV infection in the U.S. in 2011, and 5% of new HIV infections were among Americans aged 55 and older in 2010.

9. **Alcoholism and alcohol abuse are significantly greater problems in the adult population over age 65 than that under age 65.**

   False. There doesn’t appear to be substantial support for this idea. However, according to the National Survey on Drug Use and Health conducted in 2010, nearly 40% of adults age 65 and older drink alcohol. According to the survey, most of them don't have a drinking problem, but some of them drink too much. Men are more likely than women to have problems with alcohol. Research does support that older people might become more sensitive to alcohol as they age. As we grow older, our metabolism slows down so an older

person will break down alcohol more slowly than a young person and alcohol will stay in an older person's body longer. Additionally, as we age, the amount of water in the blood decreases so older adults will have a higher percentage of alcohol in their blood than younger people after drinking the same amount of alcohol. Furthermore, aging lowers the body's tolerance for alcohol which means that older adults might experience the effects of alcohol, such as lack of coordination and slurred speech, more readily than when they were younger. As older people are dealing with more chronic health conditions, oftentimes they are taking more medications. Drinking alcohol can cause certain medicines to not work properly and other medicines to become more dangerous or even deadly. Due to these issues, an older person is more susceptible to develop problems with alcohol even though his or her drinking habits have not changed.

10. Older adults have more trouble sleeping than younger adults do.

True. Older adults often experience sleep changes such as taking longer to fall asleep, frequent awakenings, daytime napping, circadian rhythm changes, lighter sleep (less time in deep sleep and REM sleep), more abnormal breathing events, and increased frequency of leg movements. The overall quality of sleep may decline with age even though more time may be spent in bed. Among the factors that may contribute to sleep problems in older adults are comorbidities, CNS disorders, GI disorders, or urinary disorders; pain; depression; polypharmacy; lack of exercise; life stressors; alcohol; smoking; environmental noises and institutional routines; and poor sleep hygiene.

11. Older adults have the highest suicide rate of any age group

False. The Centers for Disease Control & Prevention reported that in 2013 the highest suicide rate was among persons 45--64 years old (19.1/100,000). The second highest rate (18.6) occurred in those 85 years and older. The 65--84 age group had roughly the same rate as 25--44 year olds with the third highest rate. Adolescents and young adults aged 15--24 had a rate of 10.9. This is a change from the past when older adults (65+) consistently had the highest rates. Males account for the majority of suicides in all age groups.

12. High blood pressure increases with age.

True and False. There is evidence that blood pressure does increase with age. However, there is controversy over the criteria for establishing high blood pressure with increasing age. The systolic (higher number) measure is the pressure when the heart is stressed as it contracts and is recorded when the pressure cuff is first released after being tightened. The diastolic (lower number) is the blood pressure when the heart is at rest and is derived when the blood pressure returns to normal after the first rush of blood upon release of the cuff. In the general population, age 60 and older, the Eighth Report of the Joint National Commission on Detection, Evaluation and Treatment of High Blood Pressure recommends drug therapy if the systolic pressure is 90mm Hg or higher, and aims for a systolic goal of less than 150 mm Hg (150/90). The report recommends relaxing the blood pressure goals in elderly patients in order to reduce concerns related to over--treating hypertension and causing adverse events in this population that is specifically at a high risk for falls. However,

there continues to be discussion related to a cutoff of 60 years versus 80 years of age for these revised recommendations.

13. Older people perspire less, so they are more likely to suffer from hyperthermia.

True. Perspiration and quenching of thirst help to combat overheating. Older adults perspire less, are less aware of thirst and less able to feel or adapt to extremes in temperature than younger persons. Less sensitive skin sensors and less insulation of fatty deposits under the skin and the less efficient functioning of the hypothalamus (the temperature regulating mechanism in the brain) occur in older adults. Prolonged time for older adults to return to core temperature after exposure to extreme heat or cold begins at age 70 years and increases thereafter. Education and taking precautions may prevent most deaths related to temperature extremes. Increased fluid intake, gradual accommodation to climate change, rest, minimizing exertion during heat, use of fans and/or air conditioning, wearing hats and loose clothing and avoidance of alcohol are some strategies for hyperthermia.

14. All women develop osteoporosis as they age.

False. Osteoporosis ("porous bone") is associated with increasing age and is more common in women (especially White and Asian women) than men, but it is not an inevitable outcome. Gradual loss of bony tissue causes brittle bones to fracture more easily in both men and women as they age. Deficiency in bone mineral density occurs in 50% of women over 50 years to 57% of women 70 years or older, but decreases to 45% for those over 80 years. Women rarely develop osteoporosis until age 70 years. Bone mineral density (BMD) is typically measured through a DXA (dual-energy x-ray absorptiometry) test. Results are compared to the peak bone mineral density of a healthy 30-year old adult. Low bone mass that is not low enough to be diagnosed as osteoporosis is referred to as osteopenia. Prevention of osteoporosis begins with adequate calcium intake in one's teens and thereafter with increased attention to getting adequate amounts after menopause. Adequate vitamin D (from sunlight, foods, or supplements) is essential to absorbing calcium. Weight bearing exercise, hormone replacement therapy (HRT), decreased alcohol, protein, salt and caffeine consumption, and smoking cessation can also minimize bone loss. HRT may offer some protection against heart disease, cognitive impairment and bone loss, but also may present risks for cervical cancer.

15. A person's height tends to decline in old age.

True. Due to osteoporosis, osteoarthritis and a lifetime of wear and tear, upper vertebrae are weakened; joint spaces and buffering tissues wear, and muscles atrophy. These changes foster decreased padding between vertebral discs, which accounts for a loss of height. Starting at about age 40, people typically lose about .4 inch each decade and height loss may be even more rapid after age 70. The tendency to become shorter occurs among all races and in both sexes. You can help minimize loss of height by following a healthy diet, staying physically active, and preventing and treating bone loss (osteoporosis). Getting

enough calcium and vitamin D is also important to keeping bones strong. Exercises that strengthen back muscles and the body's core may be particularly beneficial. Some research has suggested that yoga may be helpful in preventing spine curvature that contributes to height loss.

16. Physical strength declines in old age.

True. Muscle mass declines, cartilage erodes, membranes fibrose (harden), and fluid thickens. These contribute to stiffness, gait problems, lessened mobility, and limited range of motion. Sarcopenia, the age-related loss of muscle mass, strength and function, starts to set in around age 45, when muscle mass begins to decline at a rate of about 1 percent a year. This gradual loss has been tied to protein deficiency, lack of exercise, and increased frailty among the elderly. Research shows that weight bearing exercise, aerobics, and weight resistance can restore muscle strength, increase stamina, stabilize balance and minimize falls.

17. Most old people lose interest in and capacity for sexual relations.

False. Sexuality, which Waite et al. (2009) define as "the dynamic outcome of physical capacity, motivation, attitudes, opportunity for partnership, and sexual conduct," exists throughout life in one form or another in everyone. It includes the physical act of intercourse as well as many other types of intimacy such as touch, hugging, and holding. Sexuality is related to overall health with those whose health is rated as excellent or good being nearly twice as likely to be sexually active as those whose health is rated as poorer. The particular form it takes varies with age and gender. In general, men are more likely than women to have a partner, more likely to be sexually active with that partner, and tend to have more positive and permissive attitudes toward sex. While the National Social Life, Health, and Aging Project showed that there was a significant decline in the percentage of men and women who reported having any sex in the preceding year (comparing 57-64, 65-74, and 75-84 years), some of this decline relates to loss of partners. Those who remained sexually active with a partner maintained remarkably constant rates of sexual activity through 65-74 and fell only modestly at the oldest ages. Normal aging physical changes in both men and women sometimes affect the ability of an older adult to have and enjoy sex. A woman's vagina may shorten and narrow and her vaginal walls become thinner and stiffer which leads to less vaginal lubrication and effects on sexual function and/or pleasure. As men age, impotence (also known as erectile dysfunction – ED) becomes more common. ED may cause a man to take longer to have an erection and it may not be as firm or large as it used to be. Additionally, the loss of erection after orgasm may happen more quickly or it may take longer before an erection is possible. Medications taken for chronic conditions such as arthritis, chronic pain, dementia, diabetes, heart disease, incontinence, stroke and depression might cause sexual problems leading to ED in men and vaginal dryness and difficulty with arousal or orgasm in women. Patient education and counseling and ability to clinically identify sexual problems can help resolve some of these issues.

18. Bladder capacity decreases with age, which leads to frequent urination.

True. Symptoms in the lower urinary tract are more prevalent among the older adults, and clinical studies have demonstrated advancing age to be associated with a reduced bladder capacity. The elastic tissue becomes tough and the bladder becomes less stretchy resulting in the bladder not holding as much urine as before. Blockage of the urethra can occur which in women is due to weakened muscles that cause the bladder or vagina to fall out of position (prolapsed). In men, the urethra can become blocked by an enlarged prostate. Aging increases the risk of kidney and bladder problems and can lead to bladder control issues such as urinary incontinence or leakage, or urinary retention which means you are not able to completely empty your bladder. Urinary tract infections (UTIs) are also common as we age as well as an increased chance for chronic kidney disease.

19. Kidney function is not affected by age.

False. The overall amount of kidney tissue decreases as well as the number of filtering units (nephrons). Nephrons filter waste material from the blood. Blood vessels supplying the kidneys can become hardened which causes the kidneys to filter blood more slowly. With aging, there is a decrease in glomerular filtration rate (GFR) and renal blood flow (RBF). The GFR is maintained at approximately 140 ml/min/1.73 m until the fourth decade. GFR declines by about 8 ml/min/1.73 per decade thereafter. Similar changes in RBF occur and it is well maintained at about 600 ml/min until approximately the fourth decade, and then declines by about 10 percent per decade. Additionally, as a person ages, the kidneys undergo a multitude of structural and functional changes. Structural changes include decreased renal mass, renal cortex and the number of glomeruli, and increased glomerular sclerosis. Aging also is associated with tubule‐interstitial fibrosis, scarring, infarction and loss of tubular mass. These structural changes are responsible for the reduced renal size of the aging kidney. Furthermore, the age related changes in the kidneys may be further complicated by concurrent comorbidities common in old age, such as hypertension, diabetes, congestive cardiac failure, atherosclerosis, urinary tract outflow obstruction, recurrent urinary tract infections and drug‐induced nephrotoxicity. It is not clear as to what extent a decline in GFR with age is physiological and what level of GFR should be considered abnormal. In summary, renal function declines physiologically with advancing age and pathologically as a result of associated diabetes and hypertension.

20. Increased problems with constipation represent a normal change as people get older.

False. Although some normal changes with aging in the gastrointestinal tract (decreased GI muscle strength and motility, lax sphincters, lowered juices) may contribute to problems with constipation, studies show little difference in colon activity of healthy older and younger people. When constipation exists, it is usually the result of factors such as inadequate exercise, a diet low in fiber, inadequate fluid intake, and certain medications. Certain health conditions can contribute to constipation—such as depression, hypothyroidism, neurological diseases like Parkinson's, or even bowel cancer. It is important to find the source of the constipation and treat it.

21. **All five senses tend to decline with age.**

    True. While there is considerable individual variation, on average sensory processes (vision, hearing, taste, smell, and touch) don't work as well as people get older. Another way to say it is that the threshold at which we take in stimuli increases with age. The eye lens, for example, is less able to change shape so as to adjust to close and far objects, and the size of the pupil narrows so as to let in less light. Hearing loss begins at age 20, and for many involves growing inability to hear higher frequencies as sensory receptors in the ear and nerve cells in the auditory pathway to the brain are lost. Taste buds become less sensitive with aging, and after age 80 more than 75 percent of older adults show major impairment in their sense of smell. Many of these normal changes can be compensated for through increasingly sophisticated assistive devices (hearing aids, glasses, etc.) and through modifications of the older person's environment.

22. **As people live longer, they face fewer acute conditions and more chronic health conditions.**

    True. The incidence of acute or temporary conditions, such as infections or the common cold, decreases with age, although those that do occur can be more debilitating and require more care. Older people are much more likely than the young to suffer from chronic conditions. These are long--term (more than three months), often permanent, and leave a residual disability that may require long--- term management or care rather than cure. More than 80% of older adults have one chronic condition, and 50% have at least two. The likelihood of multiple chronic conditions increases with age. In a Centers for Medicare and Medicaid Services report in 2012, the most common chronic conditions were high blood pressure, high cholesterol, heart disease, arthritis and diabetes in that order.

23. **Retirement is often detrimental to health-----i.e., people frequently seem to become ill or die soon after retirement.**

    False. While studies show both negative and positive correlations between retirement and health outcomes (including mortality), there is no clear evidence that retirement is actually a causal factor in health declines or mortality. The reverse is true for some as shown in the longitudinal Health and Retirement Study (HRS) where health was given as a reason for retirement among younger retirees but seldom for older retirees. With the exception of some who retire due to involuntary job loss, for most the retirement event does not appear to influence declines in either physical or mental health. Health decline is related to age or previous health problems, not retirement per se. Retirement may actually improve functional health by reducing stress on the individual. Studies based on HRS data have shown increased happiness and life satisfaction and reduced loneliness among retirees.

24. Older adults are less anxious about death than are younger and middle-aged adults.

True. Although death in industrialized society has come to be associated primarily with old age, studies generally indicate that death anxiety in adults decreases as age increases. Among the factors that may contribute to lower anxiety are a sense that goals have been fulfilled, living longer than expected, coming to terms with finitude and dealing with the deaths of friends. The general finding that older adults are less fearful of death than middle-aged counterparts should not obscure the fact that some subgroups may have considerable preoccupation and concern about death and dying. Some fear the process of dying much more than death itself.

25. People 65 years of age and older currently make up about 20% of the U.S. population.

False. According to the U.S. Census Bureau, people age 65 and older were projected to represent 14.5 percent of the total population in 2015, an increase from 12.4 percent in 2000. However, as the "baby boom" generation (born 1946-1964) is now beginning to surpass age 65, the proportion of older adults will grow dramatically. It is estimated that by 2030, adults over 65 will compose nearly 20 percent of the population.

26. Most older people are living in nursing homes.

False. A relatively small percentage of the 65+ population, 3.4% in 2013 (1.3 million) lived in institutional settings such as nursing homes. However, the percentage increases dramatically with age, ranging (in 2013) from 1% for persons 65-74 years to 3% for persons 75-84 years and 10% for persons 85+.

27. The modern family no longer takes care of its elderly.

False. Evidence from several studies and national surveys indicates that families are the major care providers for impaired older adults. Families provide 70 to 80 percent of the in-home care for older relatives with chronic impairments. Family members have cared for the typical older adult who reaches a long-term care setting for a significant amount of time first. Research has shown that adult children are the primary caregivers for older widowed women and older unmarried men, and they are the secondary caregivers in situations where the spouse of an older person is still alive. Spouses often give extensive caregiving for many years. Parent care has become a predictable and nearly universal experience across the life course, although most people are not adequately prepared for it.

28. The life expectancy of men at age 65 is about the same as that of women.

False. In 2011 remaining life expectancy at age 65 was about 2½ years less for men than women (20.22 years for women and 17.66 for men). At age 75 women's remaining life expectancy exceeds men by less than 2 years (12.76 for women and 10.94 for men). At age 85 remaining life expectancy difference is only about one year (6.87 for women and 5.81 for men). Overall life expectancy at birth is almost 5 years greater for women (80.95) than men (76.18).

29. **Remaining life expectancy of blacks at age 85 is about the same as whites.**

True. Although remaining life expectancy of blacks at age 65 is about 1.5 years less than that of whites at age 65, by the time they reach 85 remaining life expectancy is slightly higher for blacks (6.8 vs. 6.5 years). The slight excess for blacks holds for both males and females. One possible explanation for this convergence effect is that blacks who make it to the oldest ages do so in spite of many disadvantages and are "survivors" who have developed physiological and social psychological survival advantages.

30. **Social Security benefits automatically increase with inflation.**

True. Beginning in 1975 Social Security benefits are periodically automatically adjusted to inflation. Current law ties this increase to the consumer price index (CPI) or the rise in the general wage level, whichever is lower. For example, monthly Social Security and Supplemental Security Income (SSI) benefits for nearly 64 million Americans increased 1.7% in 2015.

31. **Living below or near the poverty level is no longer a significant problem for most older Americans.**

False. While the proportion of older people (65+) living below the federal poverty level declined significantly between 1959 and 2013 from 35% to 9.5%, this index rather dramatically underestimates need. The poverty level is based on an estimate of the cost of items in the Department of Agriculture's least costly nutritionally adequate food plan (assumed to be even less for a person over 65 than under 65) and multiplied by three (suggesting that food costs represent one third of a budget). This is probably not a fair representation of living costs in many areas of the country, particularly urban areas. Therefore, gerontologists and economists also look at the proportion near poverty level (anywhere from 125% to 200% of poverty level). Using 125% of the poverty level as a cut-off, another 5.6% of older adults could be considered in poverty. Those included in this group are disproportionately women, Hispanics, Blacks, those not married, and those living alone.

32. **Most older drivers are quite capable of safely operating a motor vehicle.**

True. Some older adults do have visual, motor, or cognitive impairments that make them dangerous drivers. Many drive more slowly and cautiously or avoid driving in conditions they consider threatening in order to compensate for these changes. Until approximately age 85 older adults have fewer driver fatalities per million drivers than men 20 years old, but they do have more accidents per miles driven. Unsafe speed and alcohol use are leading factors in accidents for young drivers, while right-of-way violations are the leading cause of accidents involving older drivers—which implies a breakdown in such cognitive-perceptual components as estimating the speed of oncoming cars or reacting too slowly to unexpected events. Older drivers' skills can be improved considerably by specific driver training such as through the AARP "55 ALIVE/Mature Driving" program.

33. Older workers cannot work as effectively as younger workers.

False. Negative perceptions of older workers persist because of health issues, diminished energy, discomfort with technology, closeness to retirement, and reaction to change in the work place ------ all associated with older adults. To the contrary, research identified characteristics of low turnover, less voluntary absenteeism and fewer injuries in older workers. Recent high ratings of older workers from employers cite loyalty, dependability, emotional stability, congeniality with co--workers, and consistent and accurate work outcomes. AARP gave an award in 2013 (cosponsored by the Society for Human Resource Management) to the Best Employers for Workers Over 50. Some of the nationally recognized organizations that made the list were: National Institutes of Health (NIH), Scripps Health, Atlantic Health System, Michelin North America, and the Department of Veteran Affairs–Veteran Health Administration Division.

34. Most old people are set in their ways and unable to change.

False. The majority of older people are not "set in their ways and unable to change." There is some evidence that older people tend to become more stable in their attitudes, but it is clear that older people do change. To survive, they must adapt to many events of later life such as retirement, children leaving home, widowhood, moving to new homes, and serious illness. Their political and social attitudes also tend to shift with those of the rest of society, although at a somewhat slower rate than for younger people.

35. The majority of old people are bored.

False. Older persons are involved in many and diverse activities. After retirement many participate as volunteers in churches, schools or other nonprofit organizations or engage in hobbies and other leisure pursuits. They report themselves to be "very busy." As they age most persons are likely to continue the level of activity to which they were accustomed in middle age, albeit with a different set of activities that help structure their time and provided feelings of accomplishment that were earlier provided through work and/or family responsibilities.

36. In general, most old people are pretty much alike.

False. Older adults are at least as diverse as any other age group in the population, and on many dimensions they may actually be more diverse due to their varied health, social role, and coping experiences throughout the life course. As the older population becomes more and more ethnically diverse, differences could be even greater. It is very misleading to talk about older adults as "the elderly," for this term may obscure the great heterogeneity of this age group.

37. Older adults (65+) have higher rates of criminal victimization than adults under 62 do.

False. Data from the Bureau of Justice Statistics suggest that this is not true. Statistics show that people over the age of 65 are less likely to be victims of violent crimes than younger people and this has been true for many years. Annual data from national Crime Victimization Surveys indicate that persons aged 65 and older have the lowest victimization rates of any age group in all categories, including rape, robbery, aggravated assault, and personal larceny without contact. Only for the category of personal larceny with contact (e.g., purse/wallet snatching) is the victimization rate equal to younger age groups. Nevertheless, the health and financial consequences may be greater for the older victim. It is important to note that older people are more likely to report crimes to the police than younger people so statistics are viewed as being more accurate and representative of what is actually occurring.

38. Older people tend to become more spiritual as they grow older.

True. Spirituality has to be distinguished from religion and participation in religion as a social institution (the focus of this question in the 1st edition of this quiz). Spirituality, according to Robert Atchley (2008), refers to "an inner, subjective region of life that revolves around individual experiences of being, transcending the personal self, and connecting with the sacred." It may occur in or outside of religious contexts, although cohorts born before World War II seem more likely to see the two linked than do later cohorts. Continuing to grow spiritually seems to be an especially important frontier as people move into the middle and later years. Tornstam's (2005) gerotranscendence theory asserts that we shift from a materialistic, role--oriented life philosophy to a transcendent, spiritual perspective

39. Older adults (65+) are more fearful of crime than are persons under 65.

False. Although several surveys showed that fear of crime in general exists in older adults----- despite their lower rates of victimization (the "victimization/fear paradox"), when asked about fear of specific types of crimes (e.g., murder, robbery) older people were not more fearful than those in younger age groups. Studies that have shown an increase in fear of crime in later life possibly have used measures of questionable validity. To the extent that fear does exist, it may have negative consequences for quality of life—leading to extreme measures to bar one's windows and secure doors and general hesitance to go out in the community.

40. Older people do not adapt as well as younger age groups when they relocate to a new environment.

False. While some older people may experience a period of prolonged adjustment, there is no evidence that there is special harmfulness in elderly relocation. Studies of community residents and of institutional movers have found an approximately normal distribution of outcomes ------ some positive, some negative, mostly neutral or mixed and small in degree. For

many relocation brings a better fit between personal needs and the demands of the physical and social environment. Research generally has demonstrated that adjustment to residential relocation is determined, at least in part, by perceived predictability and controllability and by the similarity between the originating and receiving environments.

41. Participation in volunteering through organizations (e.g., churches and clubs) tends to decline among older adults.

False. According to the Bureau of Labor Statistics, older adults devote many more hours to volunteering activities than middle-aged or younger adults, although there is a significant drop off after age 80. Persons who have higher levels of education, higher income, work part-time, are married, have a spouse who volunteers, have a history of volunteerism, and participate in a religious organization (since this is often the location of volunteer activities) are more likely to volunteer in later life. Research shows volunteerism to be correlated with improved self-reports of health, increased physical function, better cognitive function, reduced depressive symptoms, and longer lives.

42. Older people are much happier if they are allowed to disengage from society.

False. This view is based upon an early theory called "disengagement theory" which said that it is normal and expectable that the older person and society withdraw from each other so as to minimize the disruption caused by the older person's death. Although many people obviously do scale back certain activities, particularly if health deteriorates, there is substantial evidence that many who remain active and engaged (whether in social, family, or civic activities) have higher levels of function and happiness. For many staying involved physically, cognitively, socially, and spiritually in the social group is a basis for happiness.

43. Geriatrics is a specialty in American medicine.

True. Geriatrics refers to the clinical aspects of aging and the comprehensive health care of older persons.

Geriatrics refers to the clinical aspects of aging and the comprehensive health care of older persons. Study of geriatrics actually began in the early 1900s, although formal training in geriatrics is much more recent (the American Geriatrics Society was founded in 1942). Physicians who have completed residencies in family medicine or internal medicine can do a 12-month Geriatric Medicine Fellowship (accredited by the Accreditation Council for Graduate Medical Education). Those who are trained in Family Medicine receive a Certificate of Added Qualifications (CAQ) in Geriatric Medicine upon completion of a certification or recertification exam, whereas those in Internal Medicine taking this exam are designated as a diplomate in Geriatric Medicine by the American Board of Internal Medicine. The American Board of Psychiatry and Neurology also maintains a certification program for those specializing in geriatric psychiatry following a one-year fellowship and exam.

44. All medical schools now require students to take courses in geriatrics and gerontology.

False. As of 2010 less than half (41%) of medical schools had a structured geriatrics curriculum. In 2008 the Association of American Medical Colleges (AAMC) and the John A. Hartford Foundation developed and published a set of 26 competencies in eight general categories (medication management; cognitive and behavioral disorders; self--care capacity; falls, balance, gait disorders; health care planning and promotion; atypical presentation of disease; palliative care; hospital care for elders) that all medical students should have upon graduation. Individual schools were to determine how these competencies would be developed and evaluated. Bardach and Rowles (2012) document the barriers for inclusion of geriatric content, as well as the critical need for geriatric training in related health fields such as nursing, dentistry, pharmacy, physician assistants, physical therapy, and communication disorders. The seriousness of the situation is indicated by the fact that 27% of all physician office visits are from older adults, and other health professions report statistics as high or much higher.

45. Abuse of older adults is not a significant problem in the U.S.

False. Unfortunately, we simply do not know for certain how many people are suffering from elder abuse and neglect. It appears that female elders are abused at a higher rate than males and that the older one is, the more likely one is to be abused. Elder abuse is a significant public health problem. Each year, hundreds of thousands of adults over the age of 60 are abused, neglected, or financially exploited. In the United States alone, over 500,000 older adults are believed to be abused or neglected each year. These statistics are likely an underestimate because many victims are unable or afraid to tell the police, family, or friends about the violence. There are six types of maltreatment that occur among people over the age of 60: physical abuse, sexual abuse, emotional abuse, neglect, abandonment, and financial abuse. Older adults may be reluctant to report abuse themselves because of fear of retaliation, lack of physical and/or cognitive ability to report, or because they don't want to get the abuser (90% of whom are family members) in trouble.

46. Grandparents today take less responsibility for rearing grandchildren than ever before.

False. In 2011 around 7.7 million grandparents were living in households with their grandchildren—an increase of 23% from 2000. (U.S. Census data suggest that this increase is a long--term trend at least since 1970.) Of those approximately 3 million were the primary caregivers for their grandchildren. In the majority of families there was also a parent present even when the grandparent was the primary caregiver. Higher rates of grandparent involvement in caregiving for grandchildren appear to be associated with high divorce and teen pregnancy rates, drug and alcohol addiction, incarceration, and economic distress of adult children. Rates of grandparent involvement in childrearing are highest in Black and Asian families. There are grandparent-- headed households in every socioeconomic group, but children living with a grandmother and no parent present were most likely to be in poverty (48%).

47. Older persons take longer to recover from physical and psychological stress.

True. Older adults do experience multiple losses of loved ones and friends, illness, relocation, retirement, income, change and decline in abilities. It may take an older adult longer to adjust to a major change or recover from prolonged and intense physical and emotional stress. The recovery of an older body from a traumatic event may be delayed due to age--related decreases in cardiac output and heart rate. People who have a less effective immune system are more vulnerable to disease. However, the many older adults who have developed active and healthy lifestyles may be able to resist/mitigate some of the negative effects of stress or illness due to their physiological fitness. Likewise, coping skills that have been honed during a lifetime may lessen the damage of psychological stresses and ease adjustments to loss and change.

48. Most older adults consider their health to be good or excellent.

True. The majority of older adults consider their health to be excellent, very good, or good. Overall, most people over age 65 still rate their health positively. However, there is a pattern whereby non--Hispanic Whites typically exhibit a higher health self--rating than non--Hispanic Blacks or Hispanics. Older people make mental adjustments in their reference point of judging their own health and will typically see themselves as more healthy than they had originally expected for their age—or, compared to others their same age. Additionally, older adults are dealing with more chronic conditions that develop gradually, so they have had to adapt and compensate for them over a period of time. Oftentimes, many of these chronic conditions do not compromise their everyday functioning to a high degree, so they tend to think of their situation as being manageable and look at themselves as being healthier than a younger person might view them.

49. Older females exhibit better health care practices than older males.

True. In general women throughout adulthood are more likely to attend to minor symptoms than are men. Men are more likely to have been socialized even as children to be stoical, and consequently are less likely to see a doctor for health problems until they become clearly symptomatic. When they do get sick, they are likely to have more and longer hospital visits. Women, on the other hand, are more likely to have had regular contact with the health care system through childbirth, attending to their children's health, and having regular screening procedures for cervical and breast cancer. Although women report more chronic conditions than men in later life, the severity of their problems tends to be less than that of same age men, probably due to earlier health care interventions ------ hence the phrase "women get sicker, but men die quicker."

50. Research has shown that old age truly begins at 65.

False. Old age is a social construct. Meanings, definitions, and experiences of aging vary across cultures and throughout history. What people consider to be "old" has changed significantly just within the past 100 years in the U.S. as people live longer and healthier. Being identified as "old" is related not only to chronological age, but also to health, functional ability, social roles, and self-perception. Age 65 is an arbitrary marker that has been associated with eligibility for governmental programs such as Social Security and Medicare (although the age of eligibility for Social Security is gradually being raised to 67 by 2027)

General references

Aldwin, C. M. & Gilmer, D. F. (2013). *Health, illness, and optimal aging: Biological and psychosocial perspectives (2nd ed.)*. New York, NY: Springer.

Atchley, R. C. & Barusch, A.S. (2003). *Social forces and aging: An introduction to social gerontology* (10th ed.). Boston, MA: Cengage Learning.

Ebersole, P., Hess, P., Touhy, T., & Jett, K. (2005). *Gerontological nursing & healthy aging (2nd ed.)*. St. Louis, MO: Mosby, Inc.

Federal Interagency Forum on Aging Related Statistics. (2012). *Older Americans 2012: Key indicators of well--being.* Washington, DC: U.S. Government Printing Office.

Freedman, V.A. & Martin, L.G. (1998). Understanding trends in functional limitations among older Americans. *American Journal of Public Health.* 88 (10), 1457--1462. http://ajph.aphapublications.org/doi/pdf/10.2105/AJPH.88.10.1457

Hooyman, N. R. & Kiyak, H. A. (2007). *Social gerontology: A multidisciplinary perspective* (8th ed.). Boston, MA: Allyn and Bacon.

Maddox, G. L. (ed.). (1995). *The encyclopedia of aging* (2nd ed.). New York, NY: Springer.

Morgan, L.A., and Kunkel, S.R. (2015). *Aging, society, and the life course* (5th ed.). New York, NY: Springer.

Palmore, E. (1977). Facts on aging: A short quiz. *The Gerontologist*, 17 (4), 315--320. http://dx.doi.org/10.1093/geront/17.4.315

Palmore, E. B. (1981). The Facts on aging quiz: Part two. *The Gerontologist,* 21 (4), 431--437. http://dx.doi.org/10.1093/geront/21.4.431

Pew Research Center. (2009). *Growing old in America: Expectations vs. reality.* Retrieved from http://www.pewsocialtrends.org/files/2010/10/Getting--Old--in--America.pdf

Touhy, T. A. and Jett, K. (2012). Ebersole & Hess' *Toward healthy aging: Human needs and nursing response* (8th ed.). St. Louis, MO: Mosby, Inc.

References cited in specific answers (by question #)

1. Alzheimer's Association. (2014). 2014 *Alzheimer's disease: Facts and figures report.* Retrieved from http://www.alz.org/downloads/facts_figures_2014.pdf

8. Centers for Disease Control and Prevention. (2015). HIV *among people aged 50 and over*. Retrieved from http://www.cdc.gov/hiv/group/age/olderamericans/index.html

9. NIH Senior Health. (2015). Alcohol *use and older adults*. Retrieved from http://nihseniorhealth.gov/alcoholuse/alcoholandaging/01.html

11. American Foundation for Suicide Prevention. (2015). *Facts and figures*. Retrieved from https://www.afsp.org/understanding-suicide/facts-and-figures

12. Thomas, G., Shishehbor, M.H., Brill , D., & Nally, J.V. (2014). *New hypertension guidelines: One size fits most?* Cleveland Clinic Journal of Medicine, 81(3). Retrieved from http://www.ccjm.org/content/81/3/178.full.pdf+html

13. National Institute on Aging. (2015). Age Page: *Hyperthermia: Too hot for your health*. Retrieved from https://www.nia.nih.gov/health/publication/hyperthermia

14. National Institute on Aging. (2013). Age Page: *Osteoporosis: The bone thief*. Retrieved from https://www.nia.nih.gov/health/publication/osteoporosis

    NIH Osteoporosis and Related Bone diseases National Resource Center. (2012). Bone *mass measurement: What the numbers mean*. Retrieved from http://www.niams.nih.gov/health_info/bone/bone_health/bone_mass_measure.asp#e

15. U.S. National Library of Medicine/Medline Plus. (2012). *Aging changes in body shape.* Retrieved from http://www.nlm.nih.gov/medlineplus/ency/article/003998.htm

    Nagourney, E. (2013, Jan. 3). Why am I shrinking? *New York Times.* Retrieved from http://www.nytimes.com/2013/01/03/booming/why-am-i-getting-shorter-with-age.html

16. Scepa, C. C. & Layne, J. (2005). Low protein + low exercise = sarcopenia*. U.S. Department of Agriculture AgResearch Magazine, 53(5).* Retrieved from http://agresearchmag.ars.usda.gov/2005/may/sarco/

17. National Institute on Aging. (2013). Age Page: *Sexuality in later life.* Retrieved from https://www.nia.nih.gov/health/publication/sexuality-later-life

    Waite, L.J., Laumann, E.O., Das, A., & Schumm, L.P. (2009). Sexuality: measures of partnerships, practices, attitudes, and problems in the national social life, health, and aging study. *Journal of Gerontology: Social Sciences, 64B(S1)*, i56–i66, doi:10.1093/geronb/gbp038. Retrieved from http://www.ncbi.nlm.nih.gov/pmc/articles/PMC2763521/pdf/gbp038.pdf

18. Siroky, M. B. (2004). The aging bladder. *Reviews in urology, 6(Suppl 1): S3–S7.* Retrieved from http://www.ncbi.nlm.nih.gov/pmc/articles/PMC1472849/

19. U.S. National Library of Medicine/MedlinePlus. (2012). *Aging changes in the kidneys and bladder.* Retrieved from http://www.nlm.nih.gov/medlineplus/ency/article/004010.htm

22. Centers for Medicare and Medicaid Services. (2012). *Chronic conditions among Medicare beneficiaries: Chartbook 2012 edition.* Retrieved from http://www.cms.gov/Research--Statistics--Data--and--Systems/Statistics--Trends--and--Reports/Chronic--Conditions/Downloads/2012Chartbook.pdf

23. National Institute on Aging. (n.d.). *Growing older in America: The health and retirement study.* Retrieved from http://hrsonline.isr.umich.edu/index.php?p=dbook

25. DHHS Administration for Community Living. (2014). Administration on Aging *Projected future growth of the older population.* Retrieved from http://www.aoa.acl.gov/Aging_Statistics/future_growth/future_growth.aspx#age

26. DHHS Administration for Community Living. (2014). Administration on Aging *Profile of older Americans.* Retrieved from http://www.aoa.acl.gov/Aging_Statistics/Profile/index.aspx

28. Social Security Administration. (n.d.). *Period life table, 2011*. Retrieved from http://www.ssa.gov/oact/STATS/table4c6.html

29. Arias, E. (2014). United States life tables, 2010. *National Vital Statistics Reports, 63(7)*. Hyattsville, MD: National Center for Health Statistics.

30. Social Security Administration. (2015). Cost--*of--living adjustment (COLA) information for 2015*. Retrieved from http://www.ssa.gov/news/cola/

31. Federal Interagency Forum on Aging Related Statistics. (2012). *Older Americans 2012: Key indicators of well--being.* Washington, DC: U.S. Government Printing Office, 12. Retrieved from link at http://www.agingstats.gov/Main_Site/Data/Data_2012.aspx

    DHHS Administration for Community Living. (2014). Administration on Aging *Profile of older Americans (Poverty).* Retrieved from http://www.aoa.acl.gov/Aging_Statistics/Profile/2014/10.aspx

32. U.S. Census Bureau, Statistical Abstract of the United States (2012). *Table 1114: Licensed drivers and number in accidents by age: 2009*. Retrieved from http://www.census.gov/compendia/statab/2012/tables/12s1114.pdf

33. AARP. (2013). *Best employers for workers over 50 winners.* Retrieved from http://www.aarp.org/work/on--the--job/info--06--2013/aarp--best--employers--winners--2013.html

37. Morgan, L.A., and Kunkel, S.R. (2015). *Aging, society, and the life course* (5th ed.). New York, NY: Springer, 51.

38. Atchley, R. C. (2008). Spirituality, meaning, and the experience of aging. *Generations, 32(2)*, 12--16.

Tornstam, L. (2005). *Gerotranscendence: A developmental theory of positive aging*. New York, NY: Springer.

39. Morgan, L.A., and Kunkel, S.R. (2015). *Aging, society, and the life course* (5th ed.). New York, NY: Springer, 52.

41. Population Reference Bureau. (2011, August). Volunteering and health for aging populations. *Today's research on aging, 21.* Retrieved from http://www.prb.org/pdf11/TodaysResearchAging21.pdf

44. Bardach, S. H. & Rowles, G. D. (2012). Geriatric education in the health professions: Are we making progress? *The Gerontologist, 52(5)*, 607--618. doi:10.1093/geront/gns006

Brittain, B. (2013, Feb. 27). Many geriatrics, few geriatricians. [Weblog comment]. Retrieved from http://changingaging.org/blog/many--geriatrics--few--geriatricians/

Leipzig, R. M., Granville, L., Simpson, D., Anderson, M. B., Sauvigné, K., & Soriano, R. P. (2009). Keeping granny safe on July 1: A consensus on minimum geriatrics competencies. *Academic Medicine, 84(5), 604--610.* doi:10.1097/ACM.0b013e31819fab70

45. DHHS Administration on Aging/National Center on Elder Abuse. (n.d.). *Statistics/Data.* Retrieved from http://www.ncea.aoa.gov/Library/Data/#problem

46. Ellis, R. R. & Simmons, T. (2012). Coresident grandparents and their grandchildren: 2012. *Current Population Reports, P20--576*. Washington, DC: U.S. Census Bureau. Retrieved from http://www.census.gov/content/dam/Census/library/publications/2014/demo/p20--576.pdf

Livingston, G. (2013, Sept. 4). At grandmother's house we stay. *Pew Research Center*. Retrieved from http://www.pewsocialtrends.org/2013/09/04/at--grandmothers--house--we--stay/

48. Morgan, L.A., and Kunkel, S.R. (2015). *Aging, society, and the life course* (5th ed.). New York, NY: Springer, 211--213.
